# Comparison of Risk Scores for Lower Gastrointestinal Bleeding: A Systematic Review and Meta-analysis

**DOI:** 10.1001/jamanetworkopen.2022.14253

**Published:** 2022-05-27

**Authors:** Majed Almaghrabi, Mandark Gandhi, Leonardo Guizzetti, Alla Iansavichene, Brian Yan, Aze Wilson, Kathryn Oakland, Vipul Jairath, Michael Sey

**Affiliations:** 1 Division of Gastroenterology, Western University, London, Ontario, Canada; 2 Department of Medicine, Grand River Hospital, Kitchener, Ontario, Canada; 3 Independent Researcher, London, Ontario, Canada; 4 Library Services, London Health Sciences Centre, London, Ontario, Canada; 5 Digestive Diseases Department, HCA Healthcare UK, London, United Kingdom; 6 Lawson Health Research Institute, London Health Sciences Centre, London, Ontario, Canada; 7 Department of Epidemiology and Biostatistics, Western University, London, Ontario, Canada

## Abstract

**Importance:**

Clinical prediction models, or risk scores, can be used to risk stratify patients with lower gastrointestinal bleeding (LGIB), although the most discriminative score is unknown.

**Objective:**

To identify all LGIB risk scores available and compare their prognostic performance.

**Data Sources:**

A systematic search of Ovid MEDLINE, Embase, and the Cochrane Central Register of Controlled Trials from January 1, 1990, through August 31, 2021, was conducted. Non–English-language articles were excluded.

**Study Selection:**

Observational and interventional studies deriving or validating an LGIB risk score for the prediction of a clinical outcome were included. Studies including patients younger than 16 years or limited to a specific patient population or a specific cause of bleeding were excluded. Two investigators independently screened the studies, and disagreements were resolved by consensus.

**Data Extraction and Synthesis:**

Data were abstracted according to the Preferred Reporting Items for Systematic Reviews and Meta-analyses (PRISMA) guideline independently by 2 investigators and pooled using random-effects models.

**Main Outcomes and Measures:**

Summary diagnostic performance measures (sensitivity, specificity, and area under the receiver operating characteristic curve [AUROC]) determined a priori were calculated for each risk score and outcome combination.

**Results:**

A total of 3268 citations were identified, of which 9 studies encompassing 12 independent cohorts and 4 risk scores (Oakland, Strate, NOBLADS [nonsteroidal anti-inflammatory drug use, no diarrhea, no abdominal tenderness, blood pressure ≤100 mm Hg, antiplatelet drug use (nonaspirin), albumin <3.0 g/dL, disease score ≥2 (according to the Charlson Comorbidity Index), and syncope], and BLEED [ongoing bleeding, low systolic blood pressure, elevated prothrombin time, erratic mental status, and unstable comorbid disease]) were included in the meta-analysis. For the prediction of safe discharge, the AUROC for the Oakland score was 0.86 (95% CI, 0.82-0.88). For major bleeding, the AUROC was 0.93 (95% CI, 0.90-0.95) for the Oakland score, 0.73 (95% CI, 0.69-0.77) for the Strate score, 0.58 (95% CI, 0.53-0.62) for the NOBLADS score, and 0.65 (95% CI, 0.61-0.69) for the BLEED score. For transfusion, the AUROC was 0.99 (95% CI, 0.98-1.00) for the Oakland score and 0.88 (95% CI, 0.85-0.90) for the NOBLADS score. For hemostasis, the AUROC was 0.36 (95% CI, 0.32-0.40) for the Oakland score, 0.82 (95% CI, 0.79-0.85) for the Strate score, and 0.24 (95% CI, 0.20-0.28) for the NOBLADS score.

**Conclusions and Relevance:**

The Oakland score was the most discriminative LGIB risk score for predicting safe discharge, major bleeding, and need for transfusion, whereas the Strate score was best for predicting need for hemostasis. This study suggests that these scores can be used to predict outcomes from LGIB and guide clinical care accordingly.

## Key Points

**Question:**

Which risk score for lower gastrointestinal bleeding best discriminates safe discharge, major bleeding, need for transfusion, and need for hemostasis?

**Findings:**

In this systematic review and meta-analysis of 9 studies of 4 risk scores, the Oakland score was the most discriminative for predicting safe discharge, major bleeding, and need for transfusion, whereas the Strate score was the best at predicting need for hemostasis.

**Meaning:**

This study suggests that the Oakland and Strate scores can be used to predict lower gastrointestinal bleeding outcomes with a high degree of certainty.

## Teaser

This systematic review and meta-analysis compares 4 lower gastrointestinal bleeding risk scores based on prognostic performance.

## Introduction

Lower gastrointestinal bleeding (LGIB) is a common reason for emergency hospitalization, with an annual incidence rate upward of 87 cases per 100 000 individuals.^1,2,3,4^ Most often, LGIB is a self-limiting condition, with most cases resolving spontaneously.^1,5^ However, major bleeding resulting in blood transfusion, surgery, and even death can occur in some cases. In a large, prospective cohort study involving 2528 cases of LGIB across 143 hospitals, 26.3% of patients required blood transfusion, 1% required embolization or surgery, and 3.4% died.^5^ On a population level, LGIB is expensive and imposes considerable costs on the health care system, largely owing to hospitalizations. In a registry study of 30 498 hospitalized patients with gastrointestinal bleeding, those with LGIB had longer lengths of stay (mean [SD], 13.9 [8.8] days vs 11.6 [7.9] days) and higher resource use than patients with upper gastrointestinal bleeding.^2^

Of critical importance to the management of these patients is differentiating the majority of people who can be safely discharged for outpatient care from those who are at risk for serious adverse events and require hospitalization. Doing so would avoid the cost and burden of unnecessary hospitalization for patients at low risk of adverse outcomes while reliably identifying patients at risk for hospital admission. Clinical prediction models, or risk scores, are well suited for this purpose.^6^ A highly discriminative LGIB risk score would allow for the dichotomization of patients into high-risk and low-risk groups for adverse outcomes and can be used to guide clinical care.

To date, numerous LGIB risk scores have been developed.^7^ However, the quality of these risk scores, based in part on the representativeness of the derivation cohort, the degree of external validation, and the risk score’s accuracy, are largely unknown. Thus, the objective of this study was to conduct the first meta-analysis of LGIB risk scores, to our knowledge, based on prognostic performance.

## Methods

### Search Strategy and Study Selection

This systematic review and meta-analysis followed the Preferred Reporting Items for Systematic Reviews and Meta-analyses (PRISMA) reporting guideline^8^ and the Transparent Reporting of a Multivariable Prediction Model for Individual Prognosis or Diagnosis (TRIPOD) reporting guideline, and systematic searches were conducted in Ovid MEDLINE, Embase, and the Cochrane Central Register of Controlled Trials databases from January 1, 1990, through August 31, 2021. All non–English-language articles were excluded. The search queries were developed using a combination of subject headings and alternative free-text terms (eAppendix 1 in the Supplement). Optimized methodological search filters and text words were used to refine search results. The search strategies were modified for each database to include database-specific index terms. We also searched ClinicalTrials.gov to identify unpublished trials and abstracts from scientific meetings for the past 5 years from Digestive Disease Week, the American College of Gastroenterology Annual Scientific Meeting, and United European Gastroenterology Week. Reference lists of relevant articles and reviews were examined.

Observational studies and clinical trials with the objective of deriving or validating an LGIB risk score to predict an outcome, such as safe discharge, mortality, rebleeding, need for hemostatic intervention, and need for blood transfusions, were included. Studies including patients younger than 16 years or limited to a restricted patient population, such as older patients, or cause of bleeding, such as diverticular bleeding, were excluded. Because most studies did not directly report the number of true positives, true negatives, false positives, and false negatives, these values were calculated from extracted data; studies with insufficient information to calculate these metrics were excluded because they could not be meta-analyzed. In these situations, corresponding authors were contacted twice, 1 month apart, in an attempt to garner the missing information before the study was excluded.

Titles and abstracts were screened followed by full-text review independently by 2 of us (M.A. and M.G.). Discrepancies between the 2 reviewers were resolved by consensus, and failing that, they were resolved by a third reviewer (M.S.) who made the final determination. The study was registered in PROSPERO (CRD42018110347).

### Data Extraction and Quality Assessment

Data were extracted from eligible studies independently by 2 of us (M.A. and M.G.) and preceded by trialing of the data collection document. Disagreements were resolved by consensus, and failing that, they were resolved by a third reviewer (M.S.) who made the final determination. Data describing the study population, the risk scores used, and their diagnostic performances (true positive, true negative, false positive, or false negative for each risk score, cutoff, and outcome combination) were extracted. Studies containing more than 1 independent cohort underwent data extraction in which each cohort was denoted by a lowercase letter following the first author’s name and year of publication (eg, Strate [2005A], Strate [2005B]). Study quality was measured using the modified Quality Assessment on Diagnostic Accuracy Studies tool (QUADAS-2; eAppendix 2 in the Supplement).^9^

### Outcome Measures

Outcomes of interest for the meta-analysis included the prediction of safe discharge, major bleeding, transfusion, need for hemostasis, and mortality, although there were insufficient publications to meta-analyze the last outcome. Safe discharge was defined as the absence of major bleeding, transfusion, need for hemostasis, readmission for LGIB within 28 days, and death. Major bleeding was defined as having either *recurrent bleeding* or *severe bleeding*, as these terms were not standardized between studies but ultimately represented some form of heightened bleeding (eAppendix 3 in the Supplement). Need for hemostasis was defined as the requirement for endoscopic hemostasis, radiologic embolization, or surgery to control bleeding; radiology for diagnostic purposes and surgery for nonhemostatic purposes did not qualify. Need for blood transfusion was defined as receiving at least 1 unit of red blood cells.

The ability of LGIB risk scores to predict each outcome was measured by its discrimination, calculated based on the area under the receiver operating characteristic curve (AUROC).^8^ Discrimination is a measure of a risk score’s ability to differentiate between patients who have developed and patients who have not developed an outcome event.^6^ Thus, a highly discriminative score allows for the identification of patients who are unlikely to develop an outcome event and separate them from those who are at higher risk for the outcome event.

### Statistical Analysis

Binary study outcomes were analyzed using the random-effects regression model called the hierarchical summary receiver operating characteristic curve (HSROC) model as described by Rutter and Gatsonis.^10^ This model is useful for its connection to the bivariate-normal model with random effects^11,12^ and for producing summary receiver operating characteristic curves (ROCs) for the diagnostic test.^10^ For each outcome (safe discharge, major bleeding, transfusions, and hemostasis) and for each risk score, the HSROC model was used to explicitly model the reported risk score thresholds as fixed cofactors, allowing sensitivity and specificity to vary with threshold. These models typically require at least 3 studies for convergence. If the summary ROC models could not be fit owing to insufficient data, we attempted to use a continuity correction of one that was added to zero cells to try to improve model stability. Using the HSROC model, predicted sensitivity, specificity, and derived AUROC for a target threshold were derived. This threshold, or risk score cutoff value, was chosen based on the original publication of the risk score to predict specific LGIB-related outcomes. This approach was used because the additional information allows for more precise estimates and, in some cases, fitting a bivariate-normal regression model to the subset of studies reporting the threshold of interest resulted in unstable model estimation. There are no consensus measures or methods for quantifying or testing heterogeneity for the accuracy of diagnostic tests in meta-analyses. For selected scores and specific cutoff values, we therefore conducted a meta-analysis for each diagnostic performance measure (eg, sensitivity and specificity) individually. We used a random-effects meta-analysis model with an empirical Bayes estimator of between-study heterogeneity for the purposes of summarizing between-study heterogeneity. Although there are limitations with this approach, this model did provide familiar parameters of heterogeneity from the perspective of a meta-analysis of clinical trials. One limitation is that correlation between paired measures, such as sensitivity, specificity, and likelihood ratios, are not modeled, and, as such, these heterogeneity estimates should be interpreted cautiously. Because positive and negative predictive values depend on the prevalence of the outcome, which varied between studies, heterogeneity was not further characterized for these metrics.

The HSROC models were estimated using the PROC NLMIXED procedure in SAS, version 9.4 (SAS Institute Inc) with the MetaDAS macro published by the Cochrane Collaboration.^13^ Model parameters and summary diagnostic classification data were input to RevMan, version 5.4.1 (The Cochrane Collaboration) to produce summary ROC and forest plots.

## Results

### Literature Search and Bias Assessment

**Figure 1.**
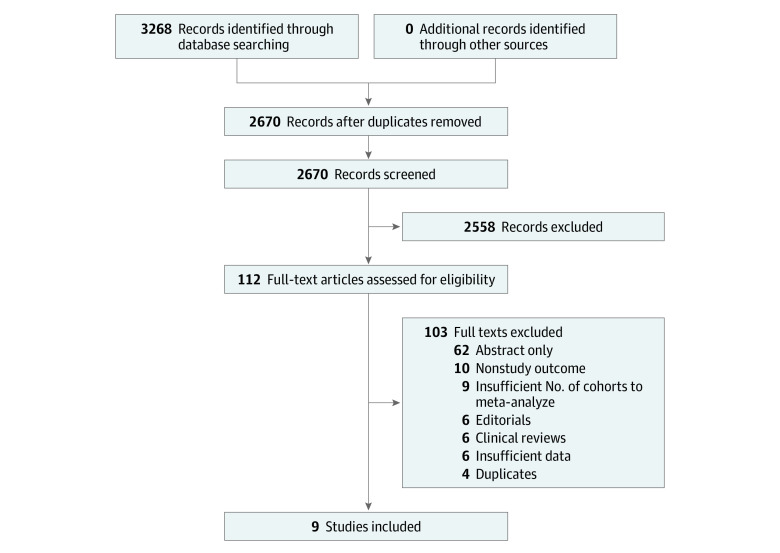
Preferred Reporting Items for Systematic Reviews and Meta-analyses Flow of Studies Through the Systematic Review

Our search identified 3268 citations; 598 were excluded because they were duplicates, and 2558 were excluded because they were not related to the derivation or validation of a LGIB risk score, leaving 112 articles for full-text review (Figure 1). From these, we identified 21 risk scores for LGIB (eAppendix 4 in the Supplement), but only 4 risk scores (Oakland, Strate, NOBLADS [nonsteroidal anti-inflammatory drug use, no diarrhea, no abdominal tenderness, blood pressure ≤100 mm Hg, antiplatelet drug use (nonaspirin), albumin <3.0 g/dL, disease score ≥2 (according to the Charlson Comorbidity Index), and syncope], and BLEED [ongoing bleeding, low systolic blood pressure, elevated prothrombin time, erratic mental status, and unstable comorbid disease] scores) were in sufficient numbers of publications for meta-analysis.^14,15,16,17,18,19,20,21,22,23,24,25,26^ Thus, the meta-analysis was performed for these 4 scores based on 9 publications^19,20,21,22,27,28,29,30,31^ encompassing 12 independent cohorts (Table^19,20,21,22,27,28,29,30,31^; eAppendices 5-8 in the Supplement).

**Table.** Characteristics of Studies Included in the Meta-analysis

Source (region)^a^	Study aim	Study design	Sample size	Age, mean (SD), y	Female, %	Underwent colonoscopy, No.	Outcomes (risk scores)
Das et al,^19^ 2003 (North America)	Validation	Prospective	70	76.5 (1.3)	49	NA	Major bleeding (BLEED score)
Strate et al,^27^ 2005A (North America)	Derivation	Retrospective	252	66 (16)	57	176	Major bleeding and hemostasis (Strate score)
Strate et al,^27^ 2005B (North America)	Validation	Prospective	275	70 (15)	55	144	Major bleeding and hemostasis (Strate score)
Ayaru et al,^20^ 2015 (Europe)	Validation	Retrospective	170	Median, 70 (range, 16-99)	47	125	Major bleeding (BLEED and Strate scores) and hemostasis (Strate score)
Aoki et al,^28^ 2016A (Asia)	Derivation	Retrospective	439	Mean, 67 (range, 18-97)	45	439	Major bleeding, hemostasis, and transfusion (NOBLADS score)
Aoki et al,^28^ 2016B (Asia)	Validation	Prospective	161	Mean, 68 (range, 16-97)	52	161	Major bleeding, hemostasis, and transfusion (NOBLADS score)
Loftus et al,^29^ 2017 (North America)	Validation	Retrospective	147	Mean, 64 (range, 61-66)	46	140	Major bleeding (Strate score)
Oakland et al,^21^ 2017A (Europe)	Derivation	Prospective	2336	68 (19)	52	NA	Safe discharge (Oakland score), major bleeding (Oakland, Strate, NOBLADS, and BLEED scores), transfusion (Oakland and NOBLADS scores), and hemostasis (Oakland, Strate and NOBLADS scores)
Oakland et al,^21^ 2017B (Europe)	Validation	Retrospective	288	66 (19)	48	NA	Safe discharge (Oakland score)
Aoki et al,^30^ 2018 (Asia)	Validation	Retrospective	511	Mean, 68.7 (range, 16-99)	34	511	Major bleeding, transfusion, and hemostasis (NOBLADS score)
Tapaskar et al,^22^ 2019 (North America)	Validation	Prospective	170	Median, 70 (range, 16-79)	58	170	Major bleeding (Oakland, Strate, and NOBLADS scores), transfusion (Oakland and NOBLADS scores), safe discharge (Oakland score), and hemostasis (Oakland, Strate, and NOBLADS scores)
Oakland et al,^31^ 2020 (North America)	Validation	Retrospective	46 128	70.1 (16.5)	50	17 896	Safe discharge, major bleeding, transfusion, and hemostasis (Oakland score)

Abbreviations: BLEED, ongoing bleeding, low systolic blood pressure, elevated prothrombin time, erratic mental status, and unstable comorbid disease; NA, not available; NOBLADS, nonsteroidal anti-inflammatory drug use, no diarrhea, no abdominal tenderness, blood pressure ≤100 mm Hg, antiplatelet drug use (nonaspirin), albumin <3.0 g/dL, disease score ≥2 (according to the Charlson Comorbidity Index), and syncope.

^a^ Where the same study is listed more than once, letters are used after the year to denote separate and independent cohorts.

The Oakland score consists of 7 clinical variables based on patient history, physical examination, and hemoglobin level and was derived using a prospective cohort involving 2336 patients from 143 hospitals in the United Kingdom.^21^ The primary intent of this risk score was to predict safe discharge, which was defined as the absence of all of the following: rebleeding, defined as additional blood transfusion requirements or a further decrease in hemoglobin concentration of 20% or more after 24 hours of clinical stability; blood transfusion; any therapeutic intervention to control bleeding; in-hospital death; and readmission with further LGIB within 28 days. Individual outcomes could also be predicted using the Oakland score. The Strate score is based on 7 clinical variables and does not require bloodwork. It was derived from a retrospective cohort of 252 patients with LGIB from a single center in the United States and was originally designed to predict severe LGIB, defined as continued bleeding within the first 24 hours of hospitalization (transfusion of ≥2 units of blood and/or hematocrit decrease ≥20%) and/or recurrent bleeding after 24 hours of stability (additional transfusions, further hematocrit decrease of ≥20%, or readmission for LGIB within 1 week of discharge), although it can also be used to predict the need for hemostasis.^32^ The NOBLADS score consists of 8 variables based on patient history, physical examination, and bloodwork and was derived from a retrospective cohort consisting of 439 patients with LGIB at a single center in Japan.^28^ The original intent was to predict severe bleeding, comprising continuous bleeding during the first 24 hours (transfusion of ≥2 units of blood and/or hematocrit decrease ≥20%) and/or recurrent bleeding after initial colonoscopy (rectal bleeding accompanied by a further decrease in hematocrit of ≥20% and/or additional blood transfusions), although it has also been studied for the prediction of the need for transfusion and the need for hemostasis. The BLEED score consists of 5 variables based on patient history, physical examination, and bloodwork and was derived from a prospective cohort of patients presenting with any gastrointestinal bleeding at 2 hospitals in the United States.^33^ The main outcome was the occurrence of any in-hospital complication, defined as either recurrent gastrointestinal hemorrhage, surgical laparotomy for hemostasis, or in-hospital mortality, although latter studies examined major bleeding as another outcome.

With the use of the QUADUS-2 tool to assess study quality, the risk for selection bias was low, as were the risks for the introduction of bias due to how the risk scores were calculated and how the outcomes were adjudicated (eFigure 1 in the Supplement). The greatest risk of bias stemmed from the flow and timing domain because most studies did not report on whether risk scores were calculated before or after outcome adjudication.

### Diagnostic Performance of LGIB Risk Scores to Predict Safe Discharge

**Figure 2.**
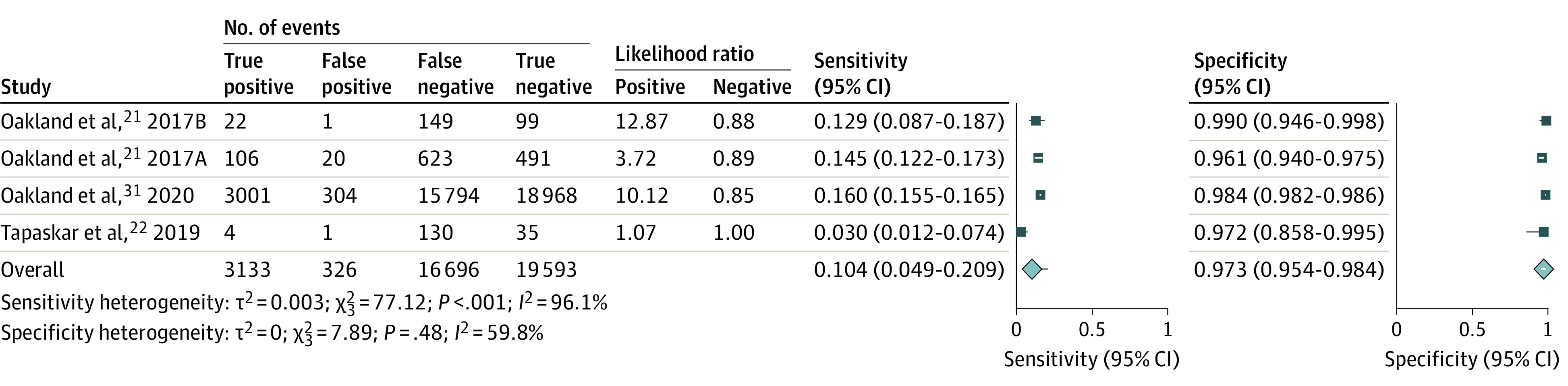
Forest Plot of Sensitivity and Specificity for the Prediction of Safe Discharge Using the Oakland Score Letters are used after the year in some studies to denote separate and independent cohorts.

Three studies containing 4 independent cohorts (n = 39 748) reported on the prediction of safe discharge using the Oakland score.^21,22,31^ Four score cutoffs were reported across these studies (≤8, ≤9, ≤10, and ≤12). All reported on the cutoff proposed in the original study of 8 or lower.^21^ The summary AUROC was 0.86 (95% CI, 0.82-0.88), and for an Oakland score of 8 or lower, the sensitivity was 10.4% (95% CI, 4.9%-20.9%) and the specificity was 97.3% (95% CI, 95.4%-98.4%) (Figure 2). None of the other risk scores were in sufficient numbers of published studies to permit meta-analysis for the outcome of safe discharge.

### Diagnostic Performance of LGIB Risk Scores to Predict Major Bleeding

Four LGIB risk scores reported on the prediction of major bleeding. These were the Oakland score, Strate score, NOBLADS score, and BLEED score.

#### Oakland Score

**Figure 3.**
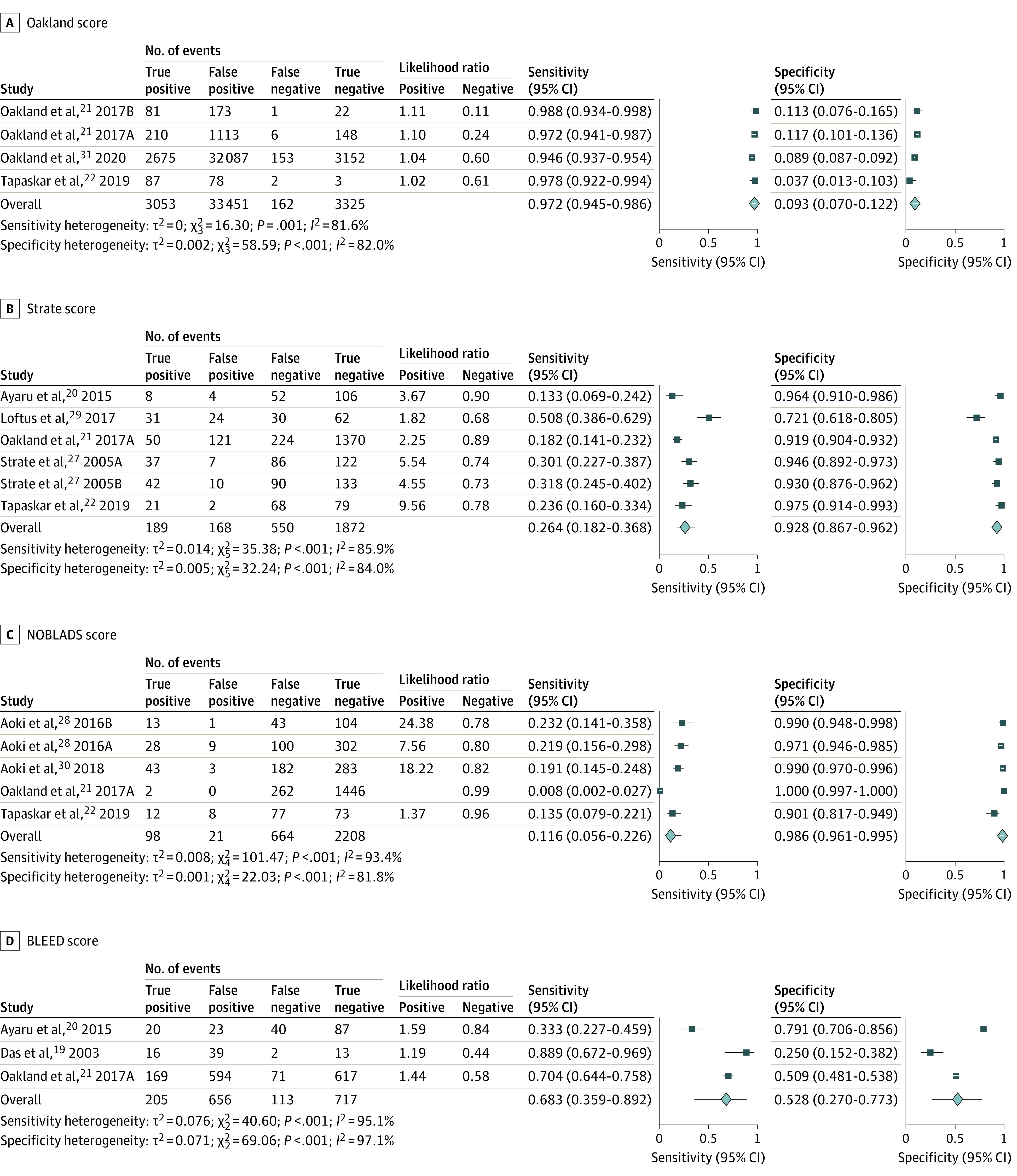
Forest Plot of Sensitivity and Specificity for the Prediction of Major Bleeding by Risk Score A, Oakland score. B, Strate score. C, NOBLADS (nonsteroidal anti-inflammatory drug use, no diarrhea, no abdominal tenderness, blood pressure ≤100 mm Hg, antiplatelet drug use [nonaspirin], albumin <3.0 g/dL, disease score ≥2 [according to the Charlson Comorbidity Index], and syncope) score. D, BLEED (ongoing bleeding, low systolic blood pressure, elevated prothrombin time, erratic mental status, and unstable comorbid disease) score. Letters are used after the year in some studies to denote separate and independent cohorts.

Three studies containing 4 independent cohorts (n = 39 991) reported on the prediction of major bleeding using the Oakland score.^21,22,31^ Across these studies, up to 4 score thresholds were reported (>8, >9, >10, and >12), with the threshold from the original study being higher than 8.^21^ The summary AUROC was 0.93 (95% CI, 0.90-0.95), and for an Oakland score higher than 8, the sensitivity was 97.2% (95% CI, 94.5%-98.6%) and the specificity was 9.3% (95% CI, 7.0%-12.2%) (Figure 3A).

#### Strate Score

Five studies containing 6 independent cohorts (n = 2779) reported on the prediction of major bleeding using the Strate score.^20,21,22,27,29^ Two score thresholds were reported (>0 and >3), with the threshold from the original study being higher than 3.^27^ The summary AUROC was 0.73 (95% CI, 0.69-0.77), and for a Strate score higher than 3, the sensitivity was 26.4% (95% CI, 18.2%-36.8%) and the specificity was 92.8% (95% CI, 86.7%-96.2%) (Figure 3B).

#### NOBLADS Score

Four studies containing 5 independent cohorts (n = 2991) reported on the prediction of major bleeding using the NOBLADS score.^21,22,28,30^ Seven total score cutoffs were reported across these studies (>0, >1, >2, >3, >4, >5, and >6), and all 4 studies reported on the original published score threshold of higher than 4.^28^ The summary AUROC curve was 0.58 (95% CI, 0.53-0.62), and for a NOBLADS score higher than 4, the sensitivity was 11.6% (95% CI, 5.6%-22.6%) and the specificity was 98.6% (95% CI, 96.1%-99.5%) (Figure 3C).

#### BLEED Score

Three studies containing 3 independent cohorts (n = 1691) reported on the prediction of major bleeding using the BLEED score.^19,20,21^ All studies reported the same score cutoff (>0) to predict the risk of major bleeding. The summary AUROC was 0.65 (95% CI, 0.61-0.69), and for a BLEED score higher than 0, the sensitivity was 68.3% (95% CI, 35.9%-89.2%) and the specificity was 52.8% (95% CI, 27.0%-77.3%) (Figure 3D).

### Diagnostic Performance of LGIB Scores to Predict Need for Transfusion

Two risk scores reported on the prediction of the need for transfusion. These were the Oakland score and the NOBLADS score.

#### Oakland Score

Three studies containing 4 independent cohorts (n = 40 138) reported on the prediction of the need for blood transfusion using the Oakland score.^21,22,31^ Across these studies, up to 4 score thresholds were reported (>8, >9, >10, and >12), with the original study using a score threshold of higher than 8.^21^ The summary AUROC was 0.99 (95% CI, 0.98-1.00), and for an Oakland score higher than 8, the sensitivity was 99.2% (95% CI, 99.1%-99.3%), and the specificity was 12.7% (95% CI, 9.1%-17.5%) (eFigure 2A in the Supplement).

#### NOBLADS Score

Four studies containing 5 independent cohorts (n = 3178) reported on the prediction of the need for transfusions using the NOBLADS score.^21,22,28,30^ Seven total score cutoffs were reported across these studies (>0, >1, >2, >3, >4, >5, and >6). All 4 studies reported on the score cutoff used in the original study of higher than 4.^28^ The summary AUROC was 0.88 (95% CI, 0.85-0.90), and for a NOBLADS score higher than 4, the sensitivity was 10.7% (95% CI, 3.6%-27.8%) and the specificity was 98.6% (95% CI, 96.8%-99.4%) (eFigure 2B in the Supplement).

### Diagnostic Performance of LGIB Scores to Predict Need for Hemostasis

Three LGIB risk scores reported on the prediction of the need for hemostasis. These were the Oakland score, the Strate score, and the NOBLADS score.

#### Oakland Score

**Figure 4.**
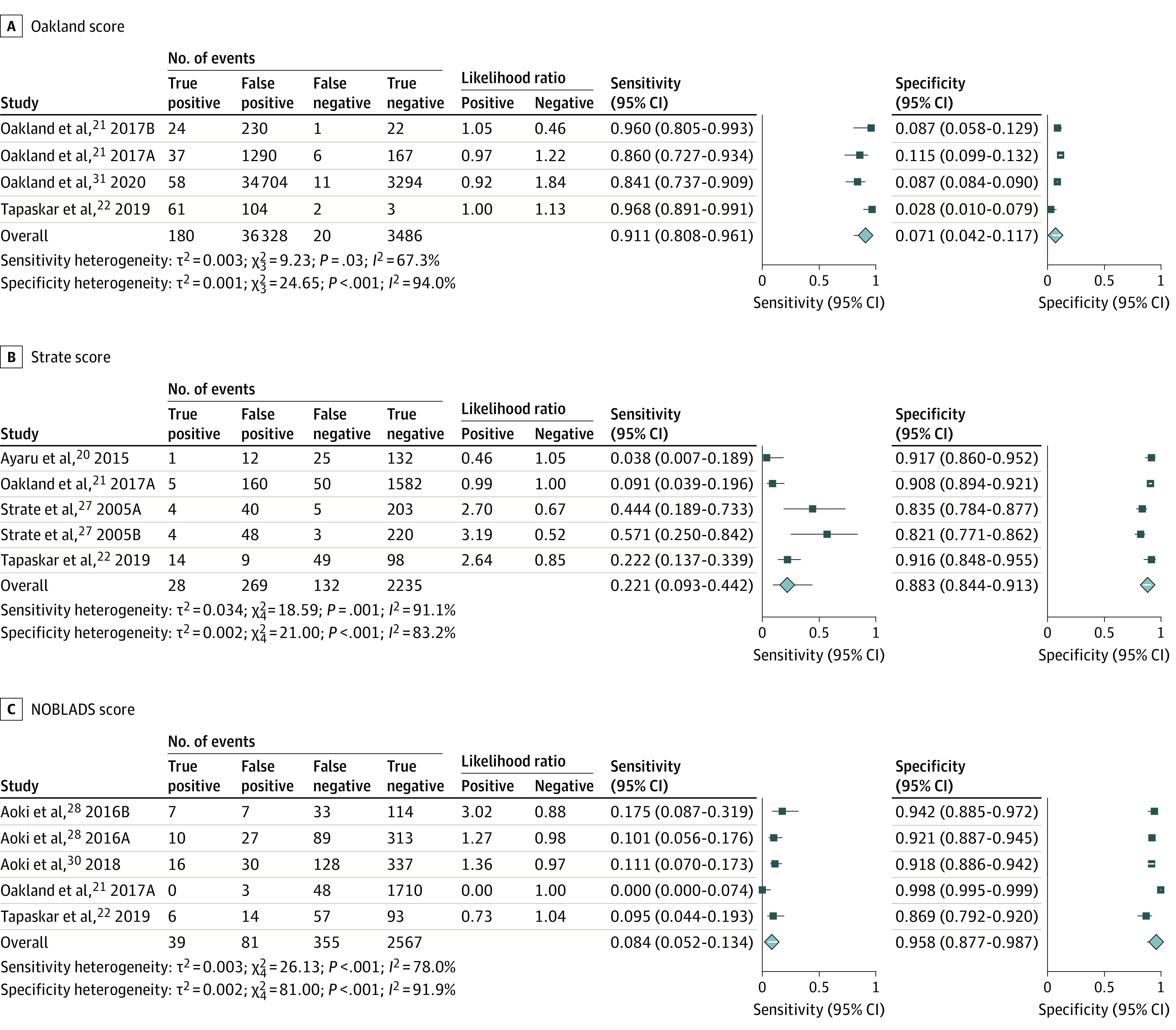
Forest Plot of Sensitivity and Specificity for the Prediction of Need for Hemostasis by Risk Score A, Oakland score. B, Strate score. C, NOBLADS (nonsteroidal anti-inflammatory drug use, no diarrhea, no abdominal tenderness, blood pressure ≤100 mm Hg, antiplatelet drug use [nonaspirin], albumin <3.0 g/dL, disease score ≥2 [according to the Charlson Comorbidity Index], and syncope) score. Letters are used after the year in some studies to denote separate and independent cohorts.

Three studies containing 4 independent cohorts (n = 40 014) reported on the prediction of the need for hemostasis using the Oakland score.^21,22,31^ Four score cutoffs were reported across the studies (>8, >9, >10, and >12), and the threshold from the original study was a score of higher than 8.^21^ The summary AUROC was 0.36 (95% CI, 0.32-0.40), and for an Oakland score higher than 8, the sensitivity was 91.1% (95% CI, 80.8%-96.1%) and the specificity was 7.1% (95% CI, 4.2%-11.7%) (Figure 4A).

#### Strate Score

Four studies containing 5 independent cohorts (n = 2664) reported on the prediction of the need for hemostasis using the Strate score.^20,21,22,27^ Two score thresholds were reported (>0 and >3), and the threshold from the original study was a score higher than 3.^27^ The summary AUROC was 0.82 (95% CI, 0.79-0.85), and for a Strate score higher than 3, the sensitivity was 22.1% (95% CI, 9.3%-44.2%) and the specificity was 88.3% (95% CI, 84.4%-91.3%) (Figure 4B).

#### NOBLADS Score

Four studies containing 5 independent cohorts (n = 3042) reported on the prediction of the need for hemostasis using the NOBLADS score.^21,22,28,30^ Seven score cutoffs were reported across these studies (>0, >1, >2, >3, >4, >5, and >6). All 4 studies reported on the score cutoff from the original study of higher than 4.^28^ The summary AUROC was 0.24 (95% CI, 0.20-0.28), and for a NOBLADS score higher than 4, the sensitivity was 8.4% (95% CI, 5.2%-13.4%) and the specificity was 95.8% (95% CI, 87.7%-98.7%) (Figure 4C).

## Discussion

In the first meta-analysis, to our knowledge, of LGIB risk prognostication models, we found the Oakland score to be the most discriminative for predicting safe discharge, major bleeding, and the need for transfusion. Of all the study outcomes examined, safe discharge is perhaps the most clinically meaningful because it can be used directly to guide patient care. This outcome is similar to the evolution of risk prognostication for upper gastrointestinal bleeding, for which risk scores originally developed to identify adverse outcomes are now used to aid discharge decision-making instead.^34,35,36^ The only risk score in the meta-analysis that predicts safe discharge was the Oakland score, which was specifically modeled to predict this outcome.^21^ The Oakland score was highly discriminative (AUROC, 0.86; 95% CI, 0.82-0.88), and when a cutoff value of 8 or lower was used, it was also highly specific for safe discharge (specificity, 97.3%; 95% CI, 95.4%-98.4%). As such, patients with LGIB who score 8 or lower using the Oakland score can be discharged for outpatient management with a high degree of certainty that they are unlikely to experience adverse outcomes in the ambulatory setting. The high specificity came at the cost of low sensitivity (10.4% [95% CI, 4.9%-20.9%]), which is actually desirable from a clinical perspective. Because specificity is defined as true negative / (true negative + false positive), a highly specific risk score would minimize the number of false-positive cases, which consists of patients who were identified as being safe for discharge but ultimately developed an adverse outcome or experienced an unsafe discharge. Conversely, a low sensitivity, which is defined as true positive / (true positive + false negative), would result in more false-negative cases, which are patients predicted to be unsafe for discharge but who ultimately did not have an adverse outcome. In balancing the need to minimize unsafe discharges and unnecessary hospitalizations, the former should take precedent over the latter, as is seen with the performance of the Oakland score.

Aside from safe discharge, other outcomes of LGIB can be predicted. These outcomes are primarily adverse events in contrast to safe discharge, which is a desirable outcome. For the outcome of major bleeding, the Oakland score was the most discriminative (AUROC, 0.93 [95% CI, 0.90-0.95]). Most patients who developed major bleeding were identified using an Oakland score higher than 8 (sensitivity, 97.2% [95% CI, 94.5%-98.6%]), although most patients in this risk group ultimately did not develop major bleeding (specificity, 9.3% [95% CI, 7.0%-12.2%]). Regardless, the prediction of major bleeding currently has only moderate clinical value because the optimal timing and choice of diagnostic and hemostatic interventions in LGIB are largely unknown, and even recommendations from recent guidelines are based largely on low-quality evidence.^1,37^ For the prediction of need for hemostasis, the Strate score performed the best (AUROC, 0.82 [95% CI, 0.79-0.85]). With a score cutoff threshold of higher than 3, the score was highly specific (88.3% [95% CI, 84.4%-91.3%]) but had a lack of sensitivity (22.1% [95% CI, 9.3%-44.2%]). Arguably, being able to predict the need for hemostasis is more useful than being able to predict major bleeding because the need for hemostasis may change clinical management. Need for transfusion was best predicted by the Oakland score (AUROC, 0.99 [95% CI, 0.98-1.00]), for which it was highly sensitive (99.2% [95% CI, 99.1%-99.3%]) but nonspecific (12.7% [95% CI, 9.1%-17.5%]). However, the accuracy of predicting need for transfusion is likely to change over time as the practice of restrictive blood transfusion for gastrointestinal bleeding becomes more widely established.^38,39^

### Limitations

There were several limitations in our meta-analysis that warrant discussion. First, we could not meta-analyze death as an outcome owing to an insufficient number of publications examining this end point. To perform a diagnostic meta-analysis, we considered scores that had at least 3 studies for each risk score and outcome combination to determine model convergence and derive summary estimates. Although we could not analyze death as an independent outcome, it was part of the composite end point of safe discharge. As such, patients at risk of death were still identified for hospitalization nonetheless. Second, there were many LGIB risk scores found in the systematic review that could not be meta-analyzed owing to an insufficient number of publications. However, these risk scores were either not validated or poorly validated compared with the 4 risk scores included in the meta-analysis, and as such, their use cannot currently be recommended. However, risk scores with promising discrimination should be further studied and compared with the Oakland and Strate scores in future studies. Third, the meta-analysis was dominated by cohorts of patients from Europe and North America. Thus, it is not clear whether the results of the present meta-analysis can be extrapolated to other populations. Fourth, all LGIB risk scores examined short-term outcomes after acute LGIB, and as such, the findings should not be extrapolated to predict long-term outcomes.

## Conclusions

In this study, the Oakland score was the most discriminative LGIB risk score for the prediction of safe discharge, major bleeding, and need for transfusion, and the Strate score was the most discriminative for the prediction of need for hemostasis. These scores can be used to predict outcomes from LGIB and guide clinical care accordingly.

## Notes

Supplement. **eAppendix 1.** Search Strategy **eAppendix 2.** QUADAS-2 Tool for Assessment for the Risk of Bias in Diagnostic Studies **eAppendix 3.** Definitions for Major Bleeding Used by Studies Included in the Meta-analysis **eAppendix 4.** LGIB Risk Scores Identified After Full Text Review but With Insufficient Numbers of Publications for Meta-analysis **eAppendix 5.** LGIB Risk Score: Oakland Score **eAppendix 6.** LGIB Risk Score: Strate Score **eAppendix 7.** LGIB Risk Score: NOBLADS Score **eAppendix 8.** LGIB Risk Score: BLEED Score **eFigure 1.** QUADAS Quality Assessment of Studies Included in the Meta-analysis **eFigure 2.** Forest Plots for Sensitivity and Specificity of Risk Scores for Need for Transfusion
