# Supplementary material for: Comparison of Risk Scores for Lower Gastrointestinal Bleeding: A Systematic Review and Meta-analysis
Source: JAMA Netw Open. 2022 May 27;5(5):e2214253. doi: 10.1001/jamanetworkopen.2022.14253 (PMC9142877; doi:10.1001/jamanetworkopen.2022.14253)
Supplement: Supplement. — eAppendix 1. Search Strategy eAppendix 2. QUADAS-2 Tool for Assessment for the Risk of Bias in Diagnostic Studies eAppendix 3. Definitions for Major Bleeding Used by Studies Included in the Meta-analysis eAppendix 4. LGIB Risk Scores Identified After Full Text Review but With Insufficient Numbers of Publications for Meta-analysis eAppendix 5. LGIB Risk Score: Oakland Score eAppendix 6. LGIB Risk Score: Strate Score eAppendix 7. LGIB Risk Score: NOBLADS Score eAppendix 8. LGIB Risk Score: BLEED Score eFigure 1. QUADAS Quality Assessment of Studies Included in the Meta-analysis eFigure 2. Forest Plots for Sensitivity and Specificity of Risk Scores for Need for Transfusion [file jamanetwopen-e2214253-s001.pdf]

Supplementary Online Content

Almaghrabi M, Gandhi M, Guizzetti L, et al. Comparison of risk scores for lower gastrointestinal bleeding: a systematic review and meta-analysis. *JAMA Netw Open.* 2022;5(5):e2214253. doi:10.1001/jamanetworkopen.2022.14253

eAppendix 1. Search Strategy
eAppendix 2. QUADAS-2 Tool for Assessment for the Risk of Bias in Diagnostic Studies
eAppendix 3. Definitions for Major Bleeding Used by Studies Included in the Meta-analysis
eAppendix 4. LGIB Risk Scores Identified After Full Text Review but With Insufficient Numbers of Publications for Meta-analysis
eAppendix 5. LGIB Risk Score: Oakland Score
eAppendix 6. LGIB Risk Score: Strate Score
eAppendix 7. LGIB Risk Score: NOBLADS Score
eAppendix 8. LGIB Risk Score: BLEED Score
eFigure 1. QUADAS Quality Assessment of Studies Included in the Meta-analysis
eFigure 2. Forest Plots for Sensitivity and Specificity of Risk Scores for Need for Transfusion

This supplementary material has been provided by the authors to give readers additional information about their work.

eAppendix 1. Search Strategy

Database: Ovid MEDLINE

1 (((gastro$ or gastric$ or gi or digestive$ or intestin$) adj2 (h?emorrhage$ or bleed$) adj5 (lower$ or LGIB)) or (lower$ adj1 GIB)).tw,kw. (2637)

2 ((diverticul$ or rectal$ or ((small$ or large$) adj2 intestin$) or colorectal$ or colon$ or bowel$) adj2 (h?emorrhage$ or bleed$)).tw,kw. (7435)

3 or/1-2 [LGIB concept] (9591)

4 exp Gastrointestinal Hemorrhage/di or assessment$.tw. or (score$ or scoring$ or (classified$ or classification$)).mp. or cl.fs. or exp "Severity of Illness Index"/ or scale$.mp. or Triage/ or triage$.mp. or APACHE.mp. (3855209)

5 exp "Sensitivity and Specificity"/ or false negative reactions/ or false positive reactions/ or diagnostic errors/ (679249)

6 ((false adj (positive$ or negative$)) or (sensitiv$ or specificity or distinguish$ or differentiat$ or enhancement or identif$ or detect$ or diagnos$ or accur$ or (predictive adj4 value$))).tw. (9373592)

7 (ROC or SROC or (receiver operat$ adj (characteristic$ or curve or analysis))).tw. (120952)

8 (or/5-7) and early.mp. (853256)

9 4 or 8 [diagnostic filter_MEDLINE] (4525593)

10 exp models, statistical/ or Prognosis/ or (predict$ or death).tw. or cohort$.mp. or prognosticat$.tw,kw. or (prognos$ adj2 (factor$ or score$ or scale$)).tw. (3611843)

11 (rebleed$ or re-bleed$ or transfusion$ or ((recur$ or re-cur$ or relaps$ or re-laps$) adj3 (h?emorrhage$ or bleed$))).mp. or risk/ or exp risk assessment/ or risk factors/ or risk$.ti. or risk$1.tw. (3015084)

12 patient admission/ or patient discharge/ or patient readmission/ or (patient$ and (admission$ or admit$ or discharge$ or readmission$ or readmit$ or re-admission$ or re-admit$)).tw,kw. (493013)

13 or/10-12 [Prognosis filter Best balance of sensitivity and specificity modified] (5880902)

14 3 and 9 and 13 (1410)

15 (((gastro$ or gastric$ or gi or digestive$) adj2 (h?emorrhage$ or bleed$)) and (score$ or scoring$ or (classified$ or classification$) or index$ or scale$ or triage$)).ti. (290)

16 (((gastro$ or gastric$ or gi or digestive$) adj2 (h?emorrhage$ or bleed$)) and (acute$ or active or severe$ or severity)).ti. (2585)

17 assessment$.tw. or (score$ or scoring$ or (classified$ or classification$)).mp. or cl.fs. or exp "Severity of Illness Index"/ or scale$.mp. or Triage/ or triage$.mp. or APACHE.mp. (3848191)

18 16 and 17 (419)

19 (15 or 18) not upper$.ti. (273)

20 14 or 19 (1622)

21 (Predictive Value of Tests/ or predict$.mp. or estimat$.tw.) and (risk/ or exp risk assessment/ or risk factors/ or risk$.mp. or (relaps$ or re-laps$ or rebleed$ or re-bleed$ or transfusion$).mp.) and

© 2022 Almaghrabi M et al. *JAMA Network Open.*

(algorithm$1 or model$ or early predict$ or decision tree$ or score$ or scoring or index$ or scale$ or triage$ or tool$1 or classified$ or classification$ or factor$).tw,kw. (520107)
22 (Prognosis/ or prognos$.mp.) and (algorithm$1 or model$ or early predict$ or decision tree$ or score$ or scoring or index$ or scale$ or triage$ or tool$1 or classified$ or classification$).ti. (46904)
23 (or/21-22) and (3 or 20) (478)
24 14 or 19 or 23 (1796)
25 limit 24 to english language (1612)
26 (pediatr$ or paediatr$ or child$ or adolescent$ or infan$ or newborn$ or boy$1 or neonat$ or prenatal$).ti. (1502705)
27 25 not 26 (1544)
28 limit 27 to "all adult (19 plus years)" (1031)
29 limit 27 to "all child (0 to 18 years)" (155)
30 27 not (29 not (28 and 29)) (1517)
31 30 not (exp Animals/ not (Human/ and exp Animals/)) (1507)
32 (mice or rat or rats or cat$1 or cattle$1 or dog$1 or goat$1 or horse$1 or rabbit$1 or sheep$1 or swine$1 or pig$1 or canine$1 or feline$1 or porcine$ or calf).ti. (1814707)
33 31 not 32 (1505)
34 limit 33 to yr="1990 -Current" (1460)
35 case reports/ or case report.ti. (2253311)
36 34 not 35 (1304)

****

Database: Embase Classic+Embase
1 (((gastro$ or gastric$ or gi or digestive$ or intestin$) adj2 (h?emorrhage$ or bleed$) adj5 (lower$ or LGIB)) or (lower$ adj1 GIB)).tw,kw. (4557)
2 ((diverticul$ or rectal$ or ((small$ or large$) adj2 intestin$) or colorectal$ or colon$ or bowel$) adj2 (h?emorrhage$ or bleed$)).tw,kw. (13790)
3 or/1-2 [LGIB concept] (17433)
4 prediction/ or predictive value/ or validation process/ or validation study/ or predict$.ti. or validat$.ti. (1191324)
5 exp blood transfusion/ or (rebleed$ or re-bleed$ or transfusion$ or ((recur$ or re-cur$ or relaps$ or re-laps$) adj3 (h?emorrhage$ or bleed$))).tw,kw. (312945)
6 hospital readmission/ or hospital discharge/ or hospital admission/ or (patient$ and (admission$ or admit$ or discharge$ or readmission$ or readmit$ or re-admission$ or re-admit$)).tw,kw. (1016677)
7 or/4-6 (2403864)
8 risk/ or exp risk assessment/ or risk factors/ or risk$1.tw. (4001169)

© 2022 Almaghrabi M et al. *JAMA Network Open.*

9 3 and 7 and 8 (1545)
10 *gastrointestinal hemorrhage/di or assessment$.tw. or (score$ or scoring$).mp. or (classified$ or classification$).tw. or exp "Severity of Illness Index"/ or scale$.tw. or triage$.tw. or APACHE.mp. or scoring system/ (4645596)
11 *"sensitivity and specificity"/ or *laboratory diagnosis/ or *prediction/ or *"prediction and forecasting"/ or *receiver operating characteristic/ or *roc curve/ or *diagnostic accuracy/ or *diagnostic value/ or *reliability/ (102375)
12 ((false adj (positive$ or negative$)) or (sensitiv$ or specificity or distinguish$ or differentiat$ or enhancement or identif$ or detect$ or diagnos$ or accur$ or (predictive adj4 value$))).tw. (12538544)
13 (ROC or SROC or (receiver operat$ adj (characteristic$ or curve or analysis))).tw. (184532)
14 (or/11-13) and early.tw. (1230282)
15 10 or 14 [diagnostic filter_EMBASE] (5608065)
16 3 and 7 and 8 and 15 (647)
17 (((gastro$ or gastric$ or gi or digestive$ or intestin$) adj2 (h?emorrhage$ or bleed$) adj5 (lower$ or LGIB)) or (lower$ adj1 GIB)).ti. (1859)
18 exp Prognosis/ or (predict$ or death).tw. or (followup or follow-up).mp. or mortality$.mp. or prognosticat$.tw,kw. or (prognos$ adj2 (factor$ or score$ or scale$)).tw. (6612933)
19 exp blood transfusion/ or (rebleed$ or re-bleed$ or transfusion$ or ((recur$ or re-cur$ or relaps$ or re-laps$) adj3 (h?emorrhage$ or bleed$))).tw. or risk/ or exp risk assessment/ or risk factors/ or risk$1.tw. (4232543)
20 hospital readmission/ or hospital discharge/ or hospital admission/ or (patient$ and (admission$ or admit$ or discharge$ or readmission$ or readmit$ or re-admission$ or re-admit$)).tw,kw. (1016677)
21 or/18-20 [Prognosis filter Best balance of sensitivity and specificity modified] (9545600)
22 15 and 17 and 21 (489)
23 16 or 22 (978)
24 (((gastro$ or gastric$ or gi or digestive$) adj2 (h?emorrhage$ or bleed$)) and (score$ or scoring$ or (classified$ or classification$) or index$ or scale$ or triage$)).ti. (636)
25 (((gastro$ or gastric$ or gi or digestive$) adj2 (h?emorrhage$ or bleed$)) and (acute$ or active or severe$ or severity)).ti. (3812)
26 assessment$.tw. or (score$ or scoring$).mp. or (classified$ or classification$).tw. or exp "Severity of Illness Index"/ or scale$.tw. or triage$.tw. or APACHE.mp. or scoring system/ (4641606)
27 25 and 26 (723)
28 (24 or 27) not upper$.ti. (434)
29 23 or 28 (1291)
30 (prediction/ or predictive value/ or validation study/ or validation process/ or predict$.tw,kw. or estimat$.tw.) and (risk/ or exp risk assessment/ or risk factors/ or risk$.mp. or (relaps$ or re-laps$

© 2022 Almaghrabi M et al. *JAMA Network Open.*

or rebleed$ or re-bleed$ or transfusion$).tw,kw.) and (algorithm$1 or model$ or early predict$ or decision tree$ or score$ or scoring or index$ or scale$ or triage$ or tool$1 or classified$ or classification$ or factor$).tw,kw. (806664)

31 (Prognosis/ or prognos$.tw.) and (algorithm$1 or model$ or early predict$ or decision tree$ or score$ or scoring or index$ or scale$ or triage$ or tool$1 or classified$ or classification$).ti. (63158)

32 (or/30-31) and (3 or 29) (1073)

33 16 or 22 or 28 or 32 (1907)

34 limit 33 to english language (1798)

35 (pediatr$ or paediatr$ or child$ or adolescent$ or infan$ or newborn$ or boy$1 or neonat$ or prenatal$).ti. (1935214)

36 34 not 35 (1763)

37 limit 36 to (adult <18 to 64 years> or aged <65+ years>) (1128)

38 limit 36 to (embryo or infant or child) (35)

39 36 not (38 not (37 and 38)) (1746)

40 39 not ((exp animal/ or nonhuman/) not exp human/) (1734)

41 (mice or rat or rats or cat$1 or cattle$1 or dog$1 or goat$1 or horse$1 or rabbit$1 or sheep$1 or swine$1 or pig$1 or canine$1 or feline$1 or porcine$ or calf).ti. (2220175)

42 40 not 41 (1733)

43 limit 42 to yr="1990 -Current" (1714)

44 case reports/ or case report.ti. (345715)

45 43 not 44 (1697)

****

Database: EBM Reviews - Cochrane Central Register of Controlled Trials

1 (((gastro$ or gastric$ or gi or digestive$ or intestin$) adj2 (h?emorrhage$ or bleed$) adj5 (lower$ or LGIB)) or (lower$ adj1 GIB)).tw,kw. (208)

2 ((diverticul$ or rectal$ or ((small$ or large$) adj2 intestin$) or colorectal$ or colon$ or bowel$) adj2 (h?emorrhage$ or bleed$)).tw,kw. (1250)

3 or/1-2 [LGIB concept] (1429)

4 exp Gastrointestinal Hemorrhage/di or assessment$.tw. or (score$ or scoring$ or (classified$ or classification$)).mp. or cl.fs. or exp "Severity of Illness Index"/ or scale$.mp. or Triage/ or triage$.mp. or APACHE.mp. (549532)

5 exp "Sensitivity and Specificity"/ or false negative reactions/ or false positive reactions/ or diagnostic errors/ (17361)

6 ((false adj (positive$ or negative$)) or (sensitiv$ or specificity or distinguish$ or differentiat$ or enhancement or identif$ or detect$ or diagnos$ or accur$ or (predictive adj4 value$))).tw. (441486)

7 (ROC or SROC or (receiver operat$ adj (characteristic$ or curve or analysis))).tw. (7202)

© 2022 Almaghrabi M et al. *JAMA Network Open.*

8 (or/5-7) and early.mp. (48569)
9 4 or 8 [diagnostic filter_MEDLINE] (577158)
10 exp models, statistical/ or Prognosis/ or (predict$ or death).tw. or cohort$.mp. or prognosticat$.tw,kw. or (prognos$ adj2 (factor$ or score$ or scale$)).tw. (242175)
11 (rebleed$ or re-bleed$ or transfusion$ or ((recur$ or re-cur$ or relaps$ or re-laps$) adj3 (h?emorrhage$ or bleed$))).mp. or risk/ or exp risk assessment/ or risk factors/ or risk$.ti. or risk$1.tw. (272470)
12 patient admission/ or patient discharge/ or patient readmission/ or (patient$ and (admission$ or admit$ or discharge$ or readmission$ or readmit$ or re-admission$ or re-admit$)).tw,kw. (71810)
13 or/10-12 [Prognosis filter Best balance of sensitivity and specificity modified] (476294)
14 3 and 9 and 13 (300)
15 (((gastro$ or gastric$ or gi or digestive$) adj2 (h?emorrhage$ or bleed$)) and (score$ or scoring$ or (classified$ or classification$) or index$ or scale$ or triage$)).ti. (25)
16 (((gastro$ or gastric$ or gi or digestive$) adj2 (h?emorrhage$ or bleed$)) and (acute$ or active or severe$ or severity)).ti. (299)
17 assessment$.tw. or (score$ or scoring$ or (classified$ or classification$)).mp. or cl.fs. or exp "Severity of Illness Index"/ or scale$.mp. or Triage/ or triage$.mp. or APACHE.mp. (549527)
18 16 and 17 (57)
19 (15 or 18) not upper$.ti. (23)
20 14 or 19 (317)
21 (Predictive Value of Tests/ or predict$.mp. or estimat$.tw.) and (risk/ or exp risk assessment/ or risk factors/ or risk$.mp. or (relaps$ or re-laps$ or rebleed$ or re-bleed$ or transfusion$).mp.) and (algorithm$1 or model$ or early predict$ or decision tree$ or score$ or scoring or index$ or scale$ or triage$ or tool$1 or classified$ or classification$ or factor$).tw,kw. (46281)
22 (Prognosis/ or prognos$.mp.) and (algorithm$1 or model$ or early predict$ or decision tree$ or score$ or scoring or index$ or scale$ or triage$ or tool$1 or classified$ or classification$).ti. (2371)
23 (or/21-22) and (3 or 20) (81)
24 14 or 19 or 23 (344)
25 limit 24 to english language (271)
26 (pediatr$ or paediatr$ or child$ or adolescent$ or infan$ or newborn$ or boy$1 or neonat$ or prenatal$).ti. (139199)
27 25 not 26 (269)
28 (mice or rat or rats or cat$1 or cattle$1 or dog$1 or goat$1 or horse$1 or rabbit$1 or sheep$1 or swine$1 or pig$1 or canine$1 or feline$1 or porcine$ or calf).ti. (4953)
29 27 not 28 (269)
30 limit 29 to yr="1990 - Current" (267)

© 2022 Almaghrabi M et al. *JAMA Network Open.*

eAppendix 2. QUADAS-2 Tool for Assessment for the Risk of Bias in Diagnostic Studies

	DOMAINS			
	**Patient selection**	**LGIB risk score**	**Assessment of adverse outcome**	**Flow and timing**
Description	Describes method of patient selection.	Describe how the LGIB risk score was conducted and interpreted	Describe the method used to ascertain whether adverse outcomes occurred.	Would anyone not get a LGIB risk score, ascertainment of adverse outcome, or get excluded from the 2x2 table?
Signaling questions<br>1 = Yes ☺<br>0 = No data 😐<br>-1= No ☹	Were consecutive patients enrolled?	Was the LGIB risk score performed without knowing if adverse outcome occurred?	Was the method used likely to correctly classify whether an adverse outcome occurred?	Was there an appropriate interval between LGIB risk score and ascertainment of adverse outcome?
	Was case-control design avoided?	Was cut-off value pre-specified *a priori*?	Was the method used done without knowledge of the LGIB risk score?	Did everyone have adverse outcome ascertained?
	Did the study avoid excessive exclusions?			Did everyone have adverse outcome ascertained in the same manner
				Was everyone included in the analysis?

© 2022 Almaghrabi M et al. *JAMA Network Open.*

Risk of bias:<br>1 = Low ☺<br>0 = No data 😐<br>-1= High ☹	Could the selection of patients have introduced bias	Could the conduct or interpretation of the LGIB risk score have introduced bias?	Could the method used have introduced bias?	Could bias have been introduced by the flow and timing of risk score and adverse outcome ascertainment?
Concerns re applicability:<br>1 = Low ☺<br>0 = No data 😐<br>-1= High ☹	Are there concerns the included patients do not match the study question?	Are there concerns that the conduct or interpretation of the LGIB risk score differed from study question?	Did the method actually measure the adverse outcome as defined by the study question?	

© 2022 Almaghrabi M et al. *JAMA Network Open.*

eAppendix 3. Definitions for Major Bleeding Used by Studies Included in the Meta-analysis

Study	Definition of Major bleeding
Das et al,[20] 2003	**Recurrent bleeding**: recurrent hematochezia after a period of 24 h of stabilization during which no active hemorrhage was observed, associated with a decrease of more than 5% in packed-cell volume or development of hemodynamic instability.
Strate et al,[28] 2005(a)	**Severe bleeding:** continued bleeding in the first 24 hours of hospitalization (defined as a RBC transfusion of ≥2 units, and/or a hematocrit decrease of ≥20%), or recurrent bleeding after 24 hours of stability (defined as more than one transfusion of RBCs, a further hematocrit decrease of ≥20%, or readmission for acute lower gastrointestinal bleeding within 1 week of discharge).
Ayaru et al,[21] 2015	**Rebleeding:** recurrent hematochezia after 24 hours of stabilization during which no active bleeding was observed, associated with any of the following as a new finding: decrease in hemoglobin of ≥2g/dl, decrease in hematocrit of ≥5%, hemodynamic instability, or having an additional RBC transfusion ≥2 units received in total).<br>**Severe bleeding:** continued bleeding in the first 24 hours of hospitalization (defined as a RBC transfusion of ≥2 units, and/or a hematocrit decrease of ≥20%), or recurrent bleeding after 24 hours of stability (defined as more than one transfusion of RBCs, a further hematocrit decrease of ≥20%, or readmission for ALGIB within 1 week of discharge).
Aoki et al,[29] 2016(a)	**Severe bleeding**: (1) continuous bleeding during the first 24 h (transfusion of 2 units of packed red blood cells and/or a decrease in hematocrit of 20%) and/or (2) recurrent bleeding after initial colonoscopy (rectal bleeding accompanied by a further decrease in hematocrit of 20% and/or additional blood transfusions).
Loftus et al,[30] 2017	**Severe bleeding:** Hematocrit decrease by 20% within 24h, ≥2 units PRBC transfused, Readmission with lower gastrointestinal bleeding within one week.
Oakland et al,[22] 2017(a)	**Rebleeding:** recurrent bleeding after 24 hours of stability (defined as more than one transfusion of RBCs, a further hematocrit decrease of >20%, or readmission for acute lower gastrointestinal bleeding within 1 week of discharge).
Aoki et al,[31] 2018	**Severe bleeding:** continuous bleeding during the first 24 hours (transfusion of ≥2 units of packed red blood cells and/or a decrease in hematocrit of ≥20%) and/or recurrent bleeding after

© 2022 Almaghrabi M et al. *JAMA Network Open.*

	initial colonoscopy (rectal bleeding accompanied by a further decrease in hematocrit of 20% or more and/or additional blood transfusions).
Tapaskar et al,[23] 2019	**In-hospital recurrent bleeding**: (1) clinically significant recurrent bleeding requiring repeat endoscopic or radiographic procedures (after initial colonoscopy) or (2) additional blood transfusion requirements or (3) a further decrease in hematocrit of 20% or more after a 24-hour period of stability after initial presentation. **Severe bleeding:** (1) continued bleeding in the first 24 hours of admission (transfusion ≥2 units of packed red blood cells and/or a decrease in hematocrit ≥20%) and/or (2) recurrent bleeding after 24 hours of clinical stability (rectal bleeding accompanied by a further decrease in hematocrit ≥20% and/or additional blood transfusions and/or readmission for LGIB within 1 week of discharge).
Oakland et al,[22] 2017	**Rebleeding:** decrease in hematocrit concentrations of 20% or more after 24 hours of clinical stability.

© 2022 Almaghrabi M et al. *JAMA Network Open.*

eAppendix 4. LGIB Risk Scores Identified After Full Text Review but With Insufficient Numbers of Publications for Meta-analysis

Author	Year	Continent	Study Aim	Study Design	Sample size	Age	%Female	Underwent colonoscopy (#)	Outcome	Risk Score
Das et al,[20] 2003	2003	North America	Derivation	Prospective	70	Mean 76.5 (SD 1.3)	49	-	Safe discharge, rebleeding, hemostatic intervention	Artificial neural network (ANN)<br><br>Multiple logistic regression (MLR)
			Validation		142	Mean 70.8 (SD 1.3	55			
Ayaru et al,[21] 2015	2015	Europe	Derivation	Retrospective	170	Median 70 (range 16-99	47	125	Rebleeding, severe bleeding and need for hemostatic intervention	Gradient Boost algorithm<br><br>Multiple logistic regression (MLR)
			Validation		130	Median 70 (range 17-101)		105		
Chong, Hill, & MacCormick,[15] 2016	2016	Australia	Derivation	Retrospective	410	Mean 58.9 (SD 18.9	49	178	Severe bleeding	HAKA
Camus et al,[16] 2016	2016	North America	Validation	Prospective	235	Mean 68 (SD 14)	32	235	Mortality, rebleeding and need for meostatic intervention	ASA, CURE, Charlson comorbidity index
Sengupta & Tapper,[17] 2017	2017	North America	Derivation	Retrospective	4044	74 (IQR 52, 75	-	-	Mortality	Sengupta
			Validation		2060					

© 2022 Almaghrabi M et al. *JAMA Network Open.*

Oakland et al,[22] 2017	2017	Europe	Validation	Retrospective	2,336	Mean 68 (SD 19)	52	-	Rebleeding, PRBC transfusion and	AIM65, GBS
Ur-Rahman et al,[18] 2018	2018	North America	Validation	Retrospective	464	Mean 66.8	-	-	Safe discharge	GBS, modified GBS
Hreinsson Sigurdardottir, Lund, & Bjornsson,[19] 2018	2018	Europe	Derivation	Retrospective	483	Mean 61 (SD 22)	46	-	Safe discharge	SHA2PE
Tapaskar et al,[23] 2019	2019	North America	Validation	Prospective	170	Median 70 (range 60-79)	58	170	Rebleeding, severe bleeding, PRBC transfusion, hemostatic intervention	GBS, AIM65, Sengupta, Charlson comorbidity index
Smith et al,[24] 2020	2020	Europe	Derivation	Retrospective	469	Median 71 (range 16-98)	-	-	Safe discharge	Birmingham
			Validation		180	Median 70 (range 19-100)				
Laursen et al,[25] 2021	2020	Europe	Validation	Retrospective	2336	Median 73 (range 29-91)	52	-	Mortality	ABC score, AIM65
Ramaekers, Perry, Leafloor, &Thirugana	2020	North America	Derivation	Retrospective	372	-	-	-	Safe discharge	Ramaekers

© 2022 Almaghrabi M et al. *JAMA Network Open.*

sambanda moorthy,[26] 2020										
Quach et al,[27] 2021	2021	Asia	Derivation	Retrospective	357	Mean 61.5	46	357	Severe bleeding	SALGIB
			Validation	Prospective	324	Mean 59.1	51	324		

© 2022 Almaghrabi M et al. *JAMA Network Open.*

eAppendix 5. LGIB risk Score: Oakland Score

**Oakland score components**		**Value**
Age, years	<40	0
	40-69	1
	≥70	2
Sex	Female	0
	Male	1
Previous lower GI bleeding admission	No	0
	Yes	1
DRE findings	No blood	0
	Blood	1
Heart rate, bpm	<70	0
	70-89	1
	90-109	2
	≥110	3
Systolic blood pressure, mmHg	50-89	5
	90-119	4
	120-129	3
	130-159	2

© 2022 Almaghrabi M et al. *JAMA Network Open.*

	≥160	0
Hemoglobin, mg/dL (g/dL)	3.6-6.9 (36-69)	22
	7.0-8.9 (70-89)	17
	9.0-10.9 (90-109)	13
	11.0-12.9 (110-129)	8
	13.0-15.9 (130-159)	4
	≥16.0 (160)	0

© 2022 Almaghrabi M et al. *JAMA Network Open.*

eAppendix 6. LGIB Risk Score: Strate Score

**Strate score components**	**Value**
Heart rate ≥ 100/min	1
Systolic blood pressure ≦ 115 mmHg	1
Syncope	1
Non tender abdominal examination	1
Aspirin (≥81 mg) use regularly for the week prior to presentation	1
Charlson Comorbidity index ≥3	1
Rectal bleeding within the first 4 h of evaluation	1

© 2022 Almaghrabi M et al. *JAMA Network Open.*

eAppendix 7. LGIB Risk Score: NOBLADS Score

**NOBLADS score components**	**Value**
No abdominal pain	1
No diarrhea	1
NSAIDs	1
Albumin < 3 g/dL	1
Systolic blood pressure ≦ 100 mmHg	1
Charlson Comorbidity index ≥2	1
Syncope	1
Antiplatelets (other than Aspirin)	1

© 2022 Almaghrabi M et al. *JAMA Network Open.*

eAppendix 8. LGIB Risk Score: BLEED Score

**BLEED score components**	**Value**
Ongoing bleeding	1
Systolic blood pressure ≦ 100 mmHg	1
Prothrombin time (PT) > 1.2	1
Erratic mental status (change in mental status)	1
Presence of unstable comorbid disease	1

© 2022 Almaghrabi M et al. *JAMA Network Open.*

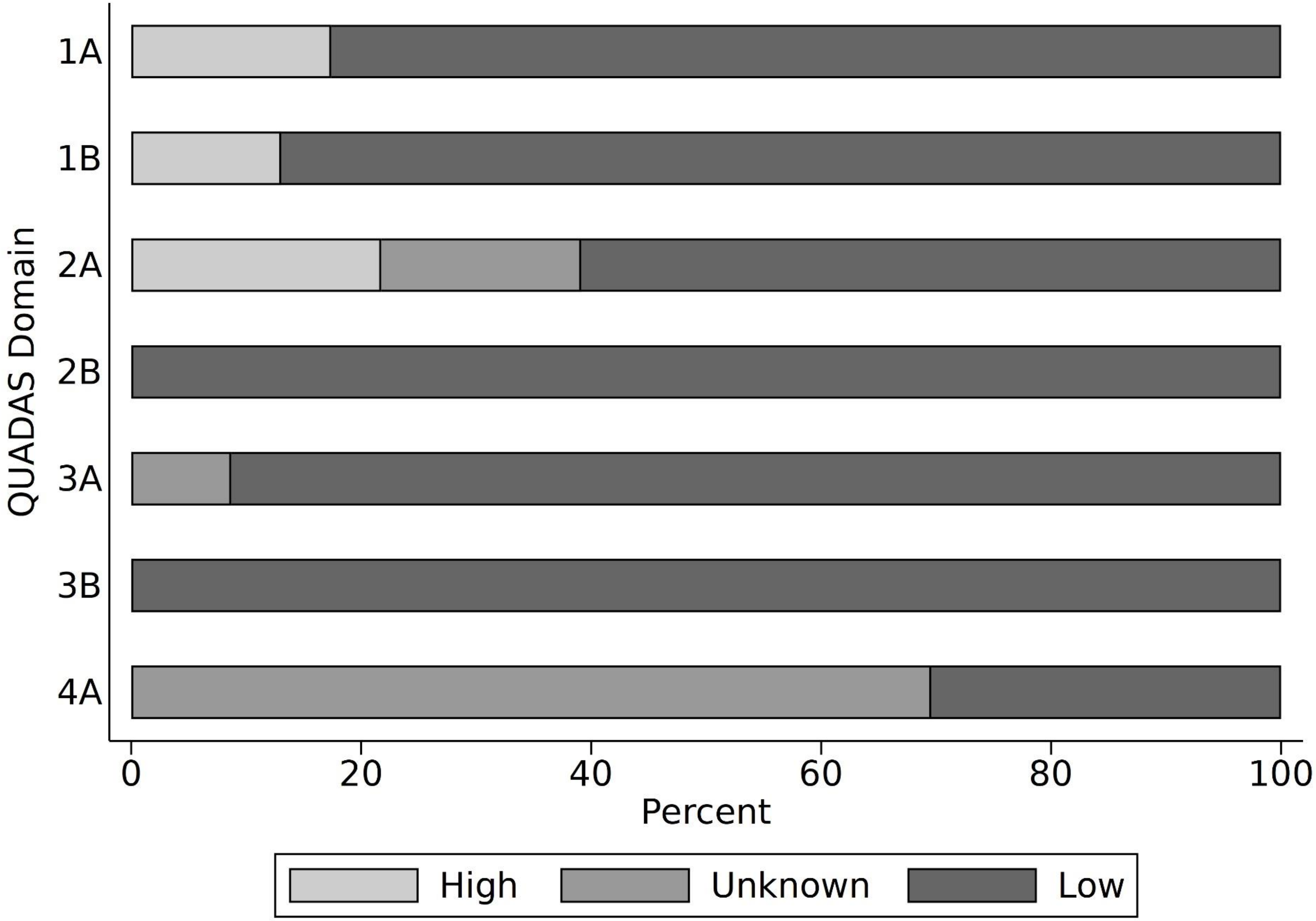

© 2022 Almaghrabi M et al. *JAMA Network Open*.

eFigure 1. QUADAS Quality Assessment of Studies Included in the Meta-analysis

Domain 1A - Patient Selection - Risk of Bias: Could the selection of patients have introduced bias?

Domain 1B - Concerns regarding applicability: Is there concern that the included patients do not match the review question?

Domain 2A - Risk Score - Risk of Bias: Could the conduct or interpretation of the risk score have introduced bias?

Domain 2B - Risk Score - Concerns regarding applicability: Is there concern that the risk score, its conduct, or interpretation differ from the review question?

Domain 3A - Outcome adjudication - Risk of Bias: Could the outcome adjudication, its conduct, or its interpretation have introduced bias?

Domain 3B - Outcome adjudication - Concerns regarding applicability: Is there concern that the target condition as defined by the outcome adjudication does not match the review question?

Domain 4A - Flow and Timing - Risk of Bias: Could the patient flow have introduced bias?

© 2022 Almaghrabi M et al. *JAMA Network Open.*

	TP	FP	FN	TN	Sensitivity (95% CI)
Oakland (2017A)	73	175	1	22	0.986 (0.927, 0.998)
Oakland (2017B)	386	1053	5	186	0.987 (0.970, 0.995)
Oakland (2020)	17110	17652	132	3173	0.992 (0.991, 0.994)
Tapaskar (2019)	103	62	2	3	0.981 (0.933, 0.995)
**Overall**	17672	18942	140	3384	0.992 (0.991, 0.993)

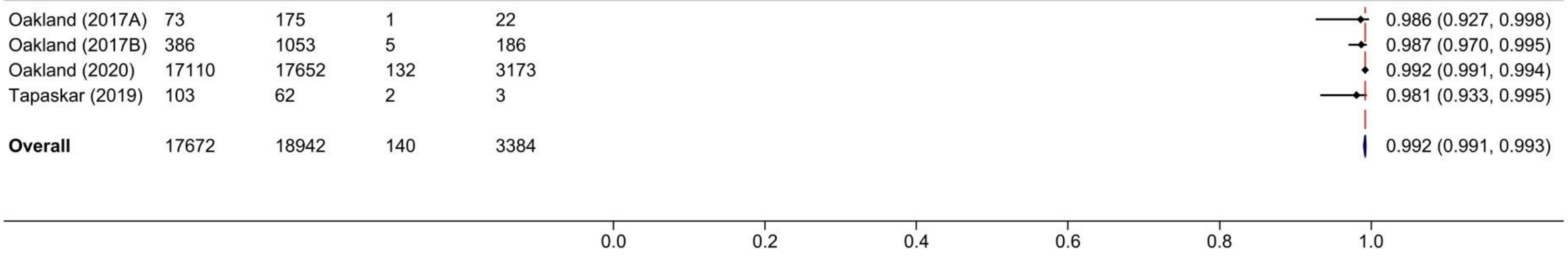

	TP	FP	FN	TN	Specificity (95% CI)
Oakland (2017A)	73	175	1	22	0.112 (0.075, 0.163)
Oakland (2017B)	386	1053	5	186	0.150 (0.131, 0.171)
Oakland (2020)	17110	17652	132	3173	0.152 (0.148, 0.157)
Tapaskar (2019)	103	62	2	3	0.046 (0.016, 0.127)
**Overall**	17672	18942	140	3384	0.127 (0.091, 0.175)

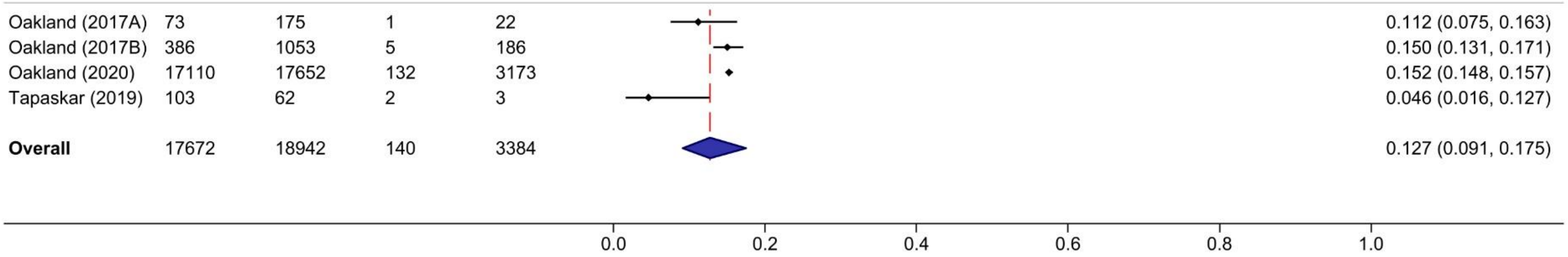

a

© 2022 Almaghrabi M et al. *JAMA Network Open*.

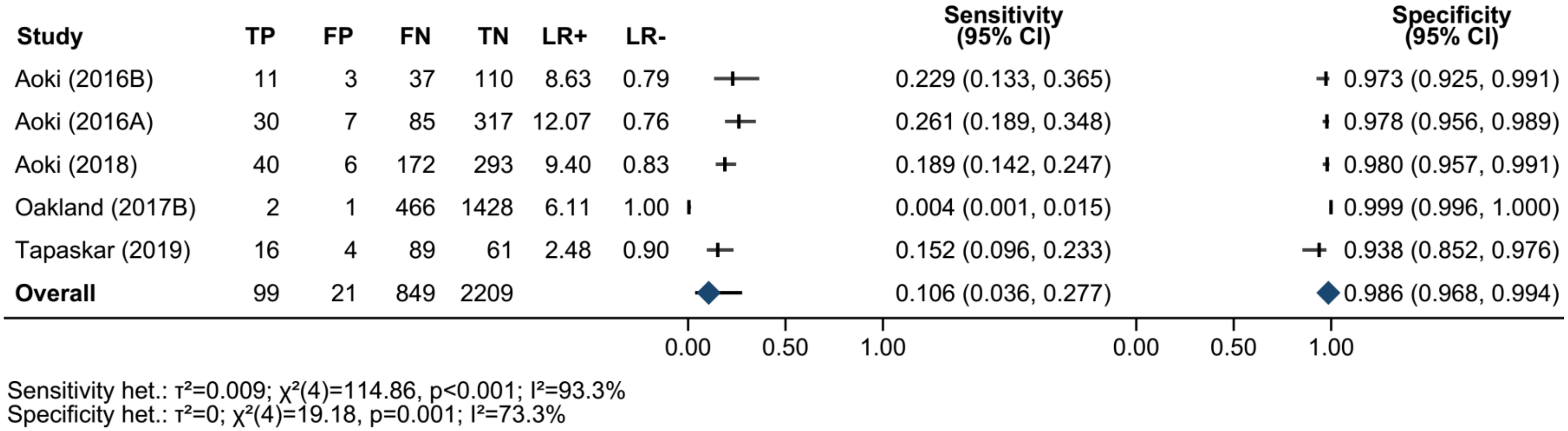

b

eFigure 2. Forest Plots for Sensitivity and Specificity of Risk Scores for Need for Transfusion
a denotes Forest plot for sensitivity and specificity of Oakland score for need for transfusion, *b* denotes Forest plot for sensitivity and specificity of NOBLADS score for need for transfusion

© 2022 Almaghrabi M et al. *JAMA Network Open.*
